# Efficacy of cognitive stimulation therapy for older adults with vascular dementia

**DOI:** 10.1590/1980-57642016dn11-040014

**Published:** 2017

**Authors:** Federica Piras, Elena Carbone, Silvia Faggian, Elisa Salvalaio, Simona Gardini, Erika Borella

**Affiliations:** 1 IRCSS Santa Lucia Foundation – Department of Clinical and Behavioral Neurology, Neuropsychiatry Laboratory Rome, Lazio, Italy.; 2 Department of General Psychology, University of Padova, Veneto, Italy.; 3 Centro Servizi Anni Sereni Scorzè (VE), Veneto, Italy.; 4 University of Parma, Italy.

**Keywords:** vascular dementia, cognitive stimulation therapy (CST), general cognitive functioning, quality of life, demência vascular, terapia de estimulação cognitiva (TSC), funcionamento cognitivo geral, qualidade de vida

## Abstract

**Background:**

Cognitive stimulation therapy (CST) is an evidence-based psychosocial intervention for people with mild-to-moderate dementia due to various etiological factors.

**Objective:**

The aim of the present study was to assess the efficacy of the CST program, Italian adaptation -CST-IT-, in individuals who have vascular dementia (VaD).

**Methods:**

Older adults with mild-to-moderate VaD (N = 35) were assigned to one of two programs: one group (N = 21) attended the 14 sessions of the CST-IT program, while the other, active control group (N = 14) took part in alternative activities. The following domains were examined: cognitive functioning, quality of life, mood, behavior, functional activities of daily living.

**Results:**

Compared with the active controls, the CST-IT group showed a greater improvement in general cognitive functioning after the intervention (i.e. score increase on the Mini-Mental State Examination and decrease on the Alzheimer's Disease Assessment Scale – Cognitive subscale). A trend towards improvement was also identified in short-term/working memory – the backward digit span task- and perceived quality of life (Quality of Life – Alzheimer's Disease scale). No significant differences emerged between the two groups for the other domains considered.

**Conclusion:**

The present results support the efficacy of CST in people with vascular dementia.

## RESUMO.

**Introdução:**

A terapia de estimulação cognitiva (TSC) é uma intervenção psicossocial baseada em evidências para pessoas com demência leve a moderada resultante de vários fatores etiológicos.

**Objetivo:**

O objetivo do presente estudo foi avaliar a eficácia do programa TSC, adaptação italiana -TSC-IT-, com indivíduos com demência vascular (DV).

**Métodos:**

Os idosos com DV leve a moderada (N = 35) foram atribuídos a um dos dois programas: um grupo (N = 21) frequentou as 14 sessões do programa TSC-IT, enquanto o outro, grupo de controle ativo (N = 14) participaram de atividades alternativas. Os seguintes domínios foram examinados: funcionamento cognitivo, qualidade de vida, humor, comportamento, atividades funcionais da vida diária.

**Resultados:**

Em comparação com os controles ativos, o grupo TSC-IT mostrou uma maior melhora no funcionamento cognitivo geral após a intervenção (ou seja, um aumento nos escores de Mini-exame do estado mental e uma diminuição na Escala de avaliação da doença de Alzheimer – subescala cognitiva). Uma tendência para uma melhoria também foi identificada na memória de curto prazo/trabalho – a tarefa de extensão de dígitos atrasados ​​– e a qualidade de vida percebida (Quality of Life - escala da doença de Alzheimer). Não houve diferenças significativas entre os dois grupos nos outros domínios considerados.

**Conclusão:**

Os resultados atuais suportam a eficácia do TSC em pessoas com demência vascular.

## INTRODUCTION

Dementia is a major global public health challenge for our generation.^1^ Options for the management of dementia include pharmacological treatments and psychosocial interventions (non-pharmacological treatments). Given the dubious results achieved to date with pharmacological therapies,^2^^,^ 3 interest in psychosocial approaches to dementia has increased considerably in recent years. Some psychosocial treatments focusing on cognitive stimulation have already proved effective.^4^ To our knowledge, cognitive stimulation therapy (CST) is the most widely used psychosocial (non-pharmacological) treatment for people with mild-to-moderate dementia,^5^ and has been studied in various countries.^6^^-^ 8 A recent review^9^ on the use of the CST program found moderate evidence of its efficacy in sustaining general cognitive functioning and promoting quality of life for people with mild-to-moderate dementia. The CST protocol, and its Italian adaptation (CST-IT),^7^ is a person-centered practice oriented towards the “personhood” of the individual suffering from dementia.^10^^,^ 11 The therapy is based on implicit learning, stimulating language and executive functioning with activities focusing on orientation, reminiscence, new ideas, thoughts, and associations to promote continuity between treatment sessions. The CST is designed to create an environment in which individuals have fun, learn, and strengthen their abilities and their social relationships with other members of a group and with the operators, preserving their social and cognitive skills for as long as possible.^12^^,^ 13

All of the studies adopting the CST protocol to date have included individuals with dementia of various etiologies, i.e. Alzheimer's disease (AD), vascular dementia (VaD), Parkinson's disease, atypical/mixed forms, and other (Lewy body dementia, mixed type dementia, Korsakov's disease), or unspecified dementia. It therefore remains to be seen whether the intervention's efficacy is dependent on the type of dementia considered.

The diagnostic criteria for VaD have been dissociated from those of AD^14^ only recently, and the term vascular cognitive impairment (VCI) has been introduced^15^ to better reflect the full range of cognitive alterations resulting from vascular factors. VaD (the most severe form of VCI) is nonetheless, the second-most-common form of dementia, and is associated with considerable morbidity and mortality rates. The construct of the vascular contribution to cognitive impairment and dementia encompasses the whole spectrum of cognitive disorders associated with all forms of vascular brain injury - and not only stroke, as was previously believed.^16^ Central to the mechanism behind the disease is the crucial role of cerebral blood vessels in keeping the brain healthy. New experimental findings have revealed a previously unrecognized functional and pathogenic synergy between neurons, glia, and vascular cells.^17^ Despite advances in our understanding, however, there are still no approved pharmacological treatments for VCI and VaD, and the development of alternative compensatory interventions remains a challenge.^3^ The heterogeneity of the cognitive disorders attributable to vascular causes points to the need to better define the VaD phenotype. Indeed, its response to cognitive interventions, as opposed to the irreversibility of the impairments and loss of independence associated with AD, may be crucially important in clarifying the clinical phenotype of VaD. The present study thus sought to ascertain the efficacy of CST-IT^7^^,^ 18 in individuals with VaD.

Taking the bottom-up approach proposed by Aguirre et al.,^13^ we conducted a single-blind, multicenter, randomized controlled trial to assess the potential benefits of the CST-IT in the following domains: (i) cognition, using traditional measures of general cognitive functioning such as the Mini-Mental State Examination and the Alzheimer's Disease Assessment Scale - Cognitive subscale, and considering more specific cognitive functions, such as short-term/working memory (measured with the backward digit span task), and language abilities (measured with the narrative language test); (ii) quality of life, using the Quality of Life – Alzheimer's Disease scale; (iii) mood and behavior, examined with the Cornell scale for depression in dementia, the social and emotional loneliness scale, and the Neuropsychiatric Inventory; and (iv) everyday life functioning, assessed with the Disability Assessment for Dementia tool.

In line with previous studies, we only expected to find improvements in the CST-IT group for traditional measures of general cognitive functioning and perceived quality of life.^9^ Changes in performance in the other dimensions were examined mainly to shed light on whether, and to what extent, the CST program could promote improvements in specific cognitive domains, such as communication skills, which are probably targeted during the intervention.^7^^,^ 19 ^,^ 20

## METHODS

### Participants.

The sample was selected from Italian residential homes for the elderly. Individuals were referred by clinicians based on the NINDS-AIREN (National Institute of Neurological Disorders and Stroke - Association Internationale pour la Recherche et l'Enseignement en Neurosciences) criteria,^21^ which encompass: 1) documented dementia (a decline in memory and in at least two other domains of intellectual ability, resulting in impaired activities of daily living); 2) evidence of cerebrovascular disorder (focal neurological signs, gait abnormalities, mood changes, psychomotor slowing, and extrapyramidal signs) in clinical history, on clinical examination, or on brain imaging (single strokes are eligible if the other criteria apply); and 3) a “reasonable relation” between dementia and cerebrovascular disorder (no early onset, a gradually worsening deficit in memory or other cognitive functions in the absence of focal lesions on neuroradiological scans). Eligibility for the CST was based on the criteria proposed by Spector et al.,^5^ but restricted to individuals with: a diagnosis of VaD in the mild to moderate range, i.e. a score of at least 14 on the Mini-Mental State Examination (MMSE);^22^ a satisfactory ability to understand and communicate; no learning disability and/or current physical illness or impairment reported in the person's clinical documents that might affect their participation; no severe behavioral or psychological symptoms of dementia that might affect their participation; no diagnosis of comorbid psychiatric disorders; and no ongoing anticholinesterase treatment.

Thirty-five eligible individuals were identified and allocated to a group that was administered the Italian version of the CST (CST-IT) (N = 21), or to an active control group (N = 14). The two groups did not differ in terms of age, gender, or years of education (Table 1). The study was performed in accordance with the Declaration of Helsinki (1964 and its later amendments).

**Table 1:** Characteristics of participants at baseline assessment by group.

	CST-IT group N = 21 (15 females)			Active control group N = 14 (13 females)		Between-group comparisons	
	M	SD		M	SD		
Age	83.81	10.93		85.43	5.18	t = –0.51	p = 0.61
Education (years)	5.90	3.22		4.64	1.69	t = 1.34	p = 0.18

CST-IT: Cognitive stimulation therapy – Italian adaptation.

### Study design.

The study was designed as a single-blind, multicenter, randomized controlled study of CST-IT for dementia.

### Materials

#### Cognitive measures

*General cognitive status.* The MMSE^22^ comprises items that test temporal and spatial orientation, immediate and delayed verbal memory, language, attention and praxis. The dependent variable was the sum of the scores (max. 30), corrected for age and education.

The Alzheimer's Disease Assessment Scale -Cognitive subscale (ADAS-Cog)^23^ consists of 11 tasks that assess orientation, memory, language, praxis, attention, and other cognitive abilities. The dependent variable was the sum of the scores (max. 70), with higher scores indicating worse functioning.

#### Memory. Short-term/working memory.

The backward digit span task^24^ entails participants listening to an increasingly long sequence of numbers (from 2 to 8) and then repeating them in the reverse order. Each level consists of two sequences of the same length. The test ends when the respondent fails to repeat both sequences. The dependent variable was the highest level of difficulty reached.

#### Language.

The narrative language test^25^ examines textual competence and discourse information content, and assesses discourse abilities as a function of the effective communication of information. Participants have to first describe a single figure (the picture “Picnic,”)^26^ and then sets of figures (two cartoon sequences).^27^ The descriptions are recorded, transcribed verbatim, and segmented using correct information unit analysis,^27^ followed by a quantitative textual analysis.^28^ The dependent variable was the sum of the items correctly and accurately reported.

#### Quality of life

The Quality of Life – Alzheimer's Disease scale (QoL-AD)^29^ is a self-report questionnaire comprising 13 items, each rated on a 4-point scale from 1 (poor) to 4 (excellent). The dependent variable was the sum of all items, where higher scores indicate better quality of life.

#### Mood

*Depression*. The Cornell scale^30^ consists of 19 items that assesses signs and symptoms of major depression in individuals with dementia. Each item is rated for severity on a scale from 0 (absent) to 2 (severe). The dependent variable was the sum of scores for the 19 items. Total scores below 6 indicate the absence of significant depressive symptoms, while those above 10 indicate probable major depression, and those above 18 indicate definite major depression.

#### Loneliness.

The 6-item social and emotional loneliness scale^31^ assesses emotional loneliness (items 1, 3, and 4), and social loneliness (items 2, 5, and 6). Each item can be scored from 1 (absolutely true) to 5 (absolutely not true). The dependent variable was the sum of scores for the 6 items. Lower scores on the social scale correspond to lower levels of social loneliness, whereas lower scores on the emotional scale indicate higher levels of emotional loneliness.

#### Behavior

The Neuropsychiatric Inventory (NPI)^32^ assesses 10 behavioral disturbances in dementia patients. The total score (frequency × severity), taken as a dependent variable, ranges from 1 to 12, where higher scores correspond to more frequent and more severe problems.

#### Activities of daily living

The Disability Assessment for Dementia (DAD)^33^^,^ 34 covers basic, instrumental and leisure activities in 10 areas, from personal hygiene to managing money and medicines. The items in each area assess the individual's ability regarding three dimensions: initiation (the ability to decide and/or start an action), planning/organization (problem-solving and decision-making), and actual performance (the ability to complete an action). The scoring options are: 1 (ability to perform the activity without help); 0 (inability to perform the activity); or N/A (activities never performed before the onset of the disease, or not performed in the past 2 weeks). The dependent variable was the total score obtained by adding the rating for each question (‘yes' = 1 point; ‘no' = 0 points) expressed as a percentage. The total number of items rated N/A were subtracted from the denominator.

#### Treatment procedure

All participants in both groups attended 18 sessions: the first two and last two were pre- and post-test individual sessions; the other 14 were group sessions during which the treatment group took part in the CST-IT program, while the active control group was involved in alternative educational activities (Table 2).

**Table 2:** Activities conducted in the CST-IT and active control groups.

• Pre-test session 1: Mini-Mental State Examination, Alzheimer's Disease Assessment Scale - Cognitive subscale, Cornell scale, Disability Assessment for Dementia, Backward digit span task. • Pre-test session 2: Neuropsychiatric Inventory, Narrative language test, Quality of Life - Alzheimer's Disease scale		
**CST-IT group Main themes/activities**		**Active control group Main themes/activities**
1	Physical games	Reading stories/newspapers, discussion
2	Sounds	Creative activities: painting, coloring, decorating, cooking.
3	My life	Reading stories/newspapers, discussion
4	Food	Creative activities: painting, coloring, decorating, cooking.
5	Current affairs	Reading stories/newspapers, discussion
6	Faces and places	Creative activities: painting, coloring, decorating, cooking.
7	Word associations	Reading stories/newspapers, discussion
8	Being creative	Creative activities: painting, coloring, decorating, cooking.
9	Categorizing objects	Reading stories/newspapers, discussion
10	Orientation	Creative activities: painting, coloring, decorating, cooking.
11	Using money	Reading stories/newspapers, discussion
12	Number games	Creative activities: painting, coloring, decorating, cooking.
13	Word games	Reading stories/newspapers, discussion
14	Team games	Creative activities: painting, coloring, decorating, cooking.
• Post-test session 1: Mini-Mental State Examination, Alzheimer's Disease Assessment Scale - Cognitive subscale, Cornell scale, Disability Assessment for Dementia, Backward digit span task. • Post-test session 2: Neuropsychiatric Inventory, Narrative language test, Quality of Life - Alzheimer's Disease scale.		

CST-IT: Cognitive stimulation therapy – Italian adaptation in which some of the original materials that did not fit with the Italian culture were changed, e.g. popular English songs were replaced with popular Italian songs, traditional English food with traditional Italian food, etc., without changing the structure and activities of the original program.

#### CST-IT intervention.^7^

The program consists of 14 structured group sessions, to be delivered twice a week for 7 weeks in small groups (7-8 people), homogeneous in terms of cognitive abilities; two trained operators act as facilitators.^5^ Each session is organized as follows: (i) Introduction: (10 min) with personalized welcome, discussion on selecting the group's name and a theme song, discussion on the day, month, year, weather, time, and the name and address of the center, using a whiteboard, discussion regarding current news, and refreshments; (ii) Main cognitive stimulation activities: (25 min), adapted to the cognitive abilities of the participants, and divided into level A (more difficult) and level B (easier) (Table 2); (iii) Conclusion (10 min): thanking everyone for attending and contributing, singing the theme song, reminding everyone the day and time of the next session, and its content, and saying goodbye.

Akin to the CST-IT group, the active control group engaged in educational activities (Table 2) for 14 sessions, again in small groups.

## RESULTS

Preliminary analyses to disclose baseline differences within each group were conducted on participants' pre-test performance for each measure of interest. The *t*-test results revealed no significant differences between the two groups at baseline. The descriptive statistics and results by group are presented in Table 3.

**Table 3:** Descriptive statistics (means and standard deviations), and Student's t-test results at baseline, for all measures of interest by group (CST-IT vs active controls).

	CST-IT group Pre-test		Active controls Pre-test		CST-IT group vs Active controls		CST-IT group Post-test		Active controls Post-test	
	M	SD	M	SD	t (33)	*p*	M	SD	M	SD
MMSE	20.29	3.42	19.03	4.65	0.92	0.36	21.72	4.45	17.79	3.47
ADAS-Cog	31.85	10.25	31.22	12.14	0.16	0.86	28.99	9.36	32.29	9.75
Backward digit span task	2.88	0.62	2.25	0.97	1.97	0.08	3.19	1.33	2.17	0.39
Narrative language test	9.90	4.56	13.69	7.19	–1.86	0.10	12.35	5.28	12.77	6.34
Quality of life – Alzheimer's Disease Scale, people with dementia	25.05	9.78	28.43	7.82	–1.08	0.27	27.35	9.41	28.00	6.87
Quality of life – Alzheimer's Disease Scale, caregivers	28.94	7.42	26.92	6.01	0.79	0.44	29.89	7.01	27.75	6.20
Social and emotional loneliness scale Total score	16.71	3.84	16.93	5.08	–0.14	0.88	18.33	5.09	18.64	4.89
Social and emotional loneliness scale: Social loneliness	8.52	2.58	8.21	3.02	0.32	0.74	8.90	3.21	8.14	3.23
Social and emotional loneliness scale: Emotional loneliness	8.19	2.18	8.71	2.84	–0.62	0.54	9.43	2.54	10.50	1.95
Cornell scale	5.90	5.45	4.14	2.54	1.13	0.27	4.19	4.33	3.50	4.01
NPI – total score	9.71	12.81	4.43	4.64	1.47	0.15	7.86	9.24	4.00	3.92
NPI – distress score	4.50	4.89	1.92	2.72	1.72	0.09	3.85	4.68	2.08	2.57
Disability Assessment for Dementia	42.33	26.45	38.44	21.61	0.36	0.72	43.10	22.63	41.92	18.98

CST-IT: Cognitive stimulation therapy – Italian adaptation; MMSE: Mini-Mental State Examination; ADAS-Cog: Alzheimer's Disease Assessment Scale – Cognitive subscale; NPI: Neuropsychiatric Inventory.

To assess the gains derived from the treatment, univariate ANCOVAs were run for each measure of interest using the post-test scores as the dependent variables, the pre-test scores as covariates, and group (CST-IT vs active control) as a between-subjects factor. The descriptive statistics for each measure of interest by group and by session are given in Table 3, and the results of the ANCOVAs are summarized in Table 4.

**Table 4:** Results of one-way ANCOVAs.

	Main effects	F (1,35)	η^2^_p_	
MMSE	covariate	40.59	0.56***	
	group	9.39	0.23**	CST-IT group > Active controls
ADAS-Cog	covariate	98.33	0.75***	
	group	5.20	0.14*	CST-IT group < Active controls
Backward digit span task	covariate	2.07	0.08	
	group	3.54	0.12^	CST group > Active controls
Narrative language test	covariate	27.05	0.47***	
	group	1.91	0.06	
Quality of life - Alzheimer's Disease Scale, people with dementia	covariate	107.01	0.77***	
	group	3.22	0.09^^	CST group > Active controls
Quality of life - Alzheimer's Disease Scale, caregivers	covariate	122.55	0.82***	
	group	<1		
Social and emotional loneliness scale: total score	covariate	68.54	0.68***	
	group	<1		
Social and emotional loneliness scale: social loneliness	covariate	48.59	0.60***	
	group	<1		
Social and emotional loneliness scale: emotional loneliness	covariate	37.98	0.54***	
	group	1.62	0.04	
Cornell scale	covariate	32.97	0.51***	
	group	<1		
NPI – total score	covariate	53.54	0.62***	
	group	4.98	0.63	
NPI – distress score	covariate	118.09	0.80***	
	group	<1		
Disability Assessment for Dementia	covariate	78.82	0.81***	
	group	<1		

* p<0.05;

** p<0.01;

*** p<0.001.

^ trend towards significance (p = 0.07);

^^ trend towards significance (p = 0.08).

MMSE: Mini-Mental State Examination; ADAS-Cog: Alzheimer's Disease Assessment Scale – Cognitive subscale; NPI: Neuropsychiatric Inventory.

In general, the analyses showed a main effect of the covariate for all the measures of interest, except the backward digit span task (Table 4).

The main effect of group was significant for the two measures of general cognitive functioning, the MMSE and the ADAS-Cog, with the CST-IT group outperforming the active controls. A trend towards an improvement for the CST-IT group, compared with the active controls, emerged for short-term/working memory, as measured by the backward digit span (*p* = 0.07), and for quality of life, as measured by the QoL-AD, in individuals with dementia only (*p* = 0.08). For all other measures, the two groups did not differ significantly (Table 4).

## DISCUSSION

After controlling for vascular risk factors (e.g. hypertension, hypercholesterolemia), the effects of pharmacological treatments for people with VaD have so far proved to be modest.^35^^,^ 36

Since the goal of evidence-based medicine is to produce reliable and relevant data for the purpose of making clinical decisions for a particular patient, and given the limited currently available evidence on the efficacy of cognitive training and cognitive rehabilitation interventions for people with mild AD or VaD, well-designed single-blind RCTs to test the effectiveness of psychosocial non-pharmacological compensatory interventions are crucial.^37^

The present study assessed the efficacy of the CST-IT program in individuals with VaD in terms of improving their cognition, including both general and specific cognitive outcome measures (i.e. short-term memory and language), and quality of life, mood, behavior and everyday life functioning. In fact, it has been suggested that the endpoints developed for studies on AD are not necessarily applicable to studies on VaD-related cognitive impairments. The primary outcome variables in VaD trials should be multidimensional and include cognition, global functioning, activities of daily living, and behavioral symptoms.^38^ In addition, given the specificity of the cognitive profile in VaD,^39^ it is important to examine the contribution of executive processes to cognition in individuals with this disorder.^38^

In line with previous studies,^9^ our results showed that the CST-IT group's general cognitive functioning improved, as measured by both the MMSE and the ADAS-Cog, immediately after completing the treatment, while functioning in the active control group did not improve. There was also a trend towards an improvement in the CST-IT group's perceived quality of life, not seen in the active controls. These findings support the efficacy of CST in sustaining the global cognitive functioning of people with dementia, also in the particular case of VaD.

A trend towards a significant improvement was also identified in the short-term/working memory domain, as measured by the backward digit span task, but not in the language domain. The task used in our study demands cognitive control abilities to actively maintain and manipulate memory representations,^40^ so the former result (the effect on active short-term memory) should be considered particularly informative regarding the efficacy of treatment in VaD. Unlike the predominant memory dysfunction in AD, the cognitive impairment that occurs in VCI and VaD is characterized by attention/concentration and executive deficits^36^^,^ 41 and the improvement seen in our participants (which might have reached statistical significance in a larger sample) on measures requiring executive attentional control suggests that the CST may target cognitive abilities specifically associated with a vascular neuropsychological impairment. When the contribution of vascular disease was not partialed out, and the program's efficacy was tested in a sample with dementia of different etiologies, the benefits of CST for short-term/working memory were not so clear. On the other hand, some studies found gains in the language domain after administering the CST,^17^^,^ 19 ^,^ 42 an outcome due mainly to the structure of the activities presented during the sessions, which specifically stimulate language skills. The dependent variable considered here was the number of correctly reported items in a referential narrative task, however, as VaD patients are not impaired on the semantic and pragmatic levels of language processing as in AD and other forms of dementia^43^, the measure adopted here may not have been sensitive enough to capture changes in our participants' communication skills.

No significant results emerged for the mood or behavior domains. It is noteworthy to mark that studies on the efficacy of CST have generally produced less consistent evidence regarding improvements in these areas as compared with findings concerning cognition.^3^^,^ 9 As expected, and in line with previous reviews,^3^^,^ 9 no changes were identified in participants' proficiency in activities of daily living as measured by the DAD, after completing the CST. Although nobody has suggested amending measures of mood and behavioral changes when considering individuals with VaD, it has been claimed that special care should be taken in assessing this group on activities of daily living because comorbidities (e.g. focal neurological signs and symptoms) resulting from the vascular disease itself might confound the issue.^38^ Moreover, the potential particular impact of VaD on executive functions needed in everyday life activities was taken into account (because the tool used here distinguishes between planning, sequencing, and completing the tasks), but functional assessments developed specifically for VCI^44^ might prove more effective in detecting treatment-induced changes in functional abilities.

While the present results support the efficacy of CST in individuals with vascular dementia, a number of limitations of this study have to be acknowledged. First, the relatively small sample size makes it difficult to generalize on the strength of our findings. Considering the variable clinical course of VCI, the potential for symptoms to improve and the non-linear progression of VaD, a follow-up and a larger sample size may be needed to conduct meaningful clinical trials. It has also been suggested^38^ that disease staging should be applied to VaD populations receiving treatment in order to address the whole range of severity of this condition, and to prove the effectiveness of a given treatment in multiple domains of the related disability. The present study is nonetheless the first to have examined the impact of this evidence-based CST intervention on this particular form of dementia.

In conclusion, our results support the utility of CST, and the importance of promoting its application and diffusion as an evidence-based intervention for sustaining general cognitive functioning (at least) in individuals with the vascular type of dementia. Since the main treatments currently available for VCI are merely preventive, the knowledge emerging from the study should orient future controlled trials on more homogeneous VCI subtypes, possibly stratified by clinical features, course of the disease, underlying pathophysiological conditions, and magnetic resonance imaging measures.
